# Fuzziness and Frustration in the Energy Landscape of Protein Folding, Function, and Assembly

**DOI:** 10.1021/acs.accounts.0c00813

**Published:** 2021-02-08

**Authors:** Stefano Gianni, María Inés Freiberger, Per Jemth, Diego U. Ferreiro, Peter G. Wolynes, Monika Fuxreiter

**Affiliations:** † Istituto Pasteur - Fondazione Cenci Bolognetti, Dipartimento di Scienze Biochimiche “A. Rossi Fanelli” and Istituto di Biologia e Patologia Molecolari del CNR, Sapienza Università di Roma, 00185 Rome, Italy; ‡ Protein Physiology Lab, Departamento de Química Biológica, Facultad de Ciencias Exactas y Naturales, Universidad de Buenos Aires-CONICET-IQUIBICEN, 1428 Buenos Aires, Argentina; § Department of Medical Biochemistry and Microbiology, Uppsala University, Husargatan 3, SE-75123 Uppsala, Sweden; ∥ Center for Theoretical Biological Physics, Rice University, 6500 Main Street, Houston, Texas 77251-1892, United States; ⊥ MTA-DE Laboratory of Protein Dynamics, Department of Biochemistry and Molecular Biology, University of Debrecen, Nagyerdei krt 98, H-4032 Debrecen, Hungary; # Department of Biomedical Sciences, University of Padova, Via Ugo Bassi 58/B, 35131 Padova, Italy

## Abstract

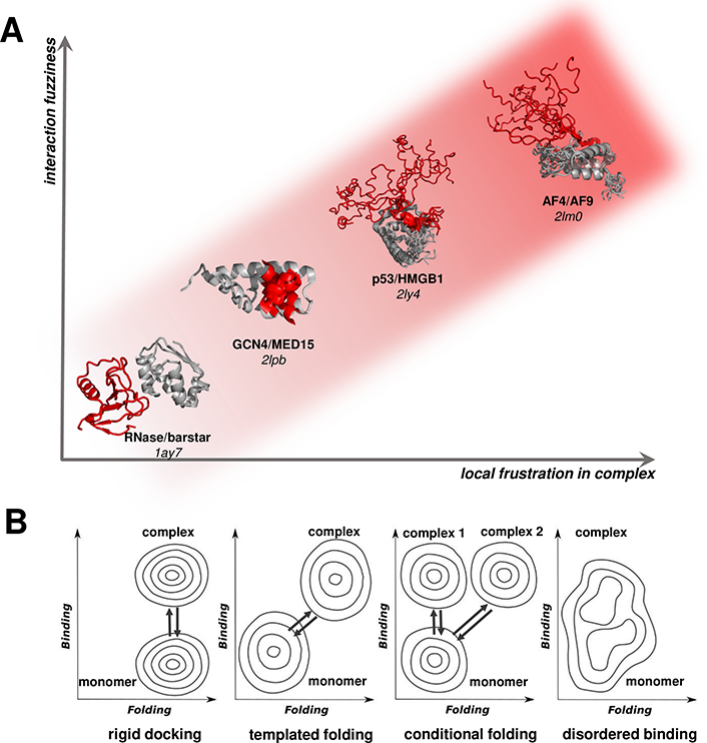

Are all protein interactions fully optimized? Do suboptimal interactions compromise specificity? What is the functional impact of frustration? Why does evolution not optimize some contacts? Proteins and their complexes are best described as ensembles of states populating an energy landscape. These ensembles vary in breadth from narrow ensembles clustered around a single average X-ray structure to broader ensembles encompassing a few different functional “taxonomic” states on to near continua of rapidly interconverting conformations, which are called “fuzzy” or even “intrinsically disordered”. Here we aim to provide a comprehensive framework for confronting the structural and dynamical continuum of protein assemblies by combining the concepts of energetic frustration and interaction fuzziness. The diversity of the protein structural ensemble arises from the frustrated conflicts between the interactions that create the energy landscape. When frustration is minimal after folding, it results in a narrow ensemble, but residual frustrated interactions result in fuzzy ensembles, and this fuzziness allows a versatile repertoire of biological interactions. Here we discuss how fuzziness and frustration play off each other as proteins fold and assemble, viewing their significance from energetic, functional, and evolutionary perspectives.

We demonstrate, in particular, that the common physical origin of both concepts is related to the ruggedness of the energy landscapes, intramolecular in the case of frustration and intermolecular in the case of fuzziness. Within this framework, we show that alternative sets of suboptimal contacts may encode specificity without achieving a single structural optimum. Thus, we demonstrate that structured complexes may not be optimized, and energetic frustration is realized via different sets of contacts leading to multiplicity of specific complexes. Furthermore, we propose that these suboptimal, frustrated, or fuzzy interactions are under evolutionary selection and expand the biological repertoire by providing a multiplicity of biological activities. In accord, we show that non-native interactions in folding or interaction landscapes can cooperate to generate diverse functional states, which are essential to facilitate adaptation to different cellular conditions. Thus, we propose that not fully optimized structures may actually be beneficial for biological activities of proteins via an alternative set of suboptimal interactions. The importance of such variability has not been recognized across different areas of biology.

This account provides a modern view on folding, function, and assembly across the protein universe. The physical framework presented here is applicable to the structure and dynamics continuum of proteins and opens up new perspectives for drug design involving not fully structured, highly dynamic protein assemblies.

## KEY REFERENCES

FuxreiterM.Fuzziness in Protein Interactions-A Historical Perspective. J. Mol. Biol.2018, 430, 2278–2287.2947733710.1016/j.jmb.2018.02.015(1)*10-years overview of the fuzziness concept and application areas*.

ChenM.; ChenX.; SchaferN. P.; ClementiC.; KomivesE. A.; FerreiroD. U.; WolynesP. G.Surveying biomolecular frustration at atomic resolution. Nat. Commun.2020, 11, 5944.3323015010.1038/s41467-020-19560-9PMC7683549(2)*A comprehensive overview of the frustration concept and its role in shaping protein function*.

FreibergerM. I.; WolynesP. G.; FerreiroD. U.; FuxreiterM.Frustration in protein complexes leads to interaction versatility. 2020, BioRxiv. https://www.biorxiv.org/content/10.1101/2020.11.11.378091v1.10.1021/acs.jpcb.0c11068PMC804130933667107(3)*Systematic analysis of 160 fuzzy complexes demonstrates a high degree of frustration and reveals the origin of specificity without an optimal structure*.

JemthP.; KarlssonE.; VogeliB.; GuzovskyB.; AnderssonE.; HultqvistG.; DoganJ.; GuntertP.; RiekR.; ChiC. N.Structure and dynamics conspire in the evolution of affinity between intrinsically disordered proteins. Sci. Adv.2018, 4, eaau4130.3039765110.1126/sciadv.aau4130PMC6200366(4)*Biophysical analysis showing the interplay among plasticity, structure, and dynamics during evolution of an interaction involving disordered proteins*.

## 1 Introduction

The crystallographers’ paradigm that function follows structure has played a pivotal role in protein science. Yet, the puzzle of how oxygen could get into a rigid hemoglobin arose as soon as its structure was determined. Proteins can be understood only when their motions are taken into account.^5^ The molecular movements and rearrangements needed for function range from ps side-chain rotations to subunit and domain rearrangements on the ms time scale and on to the most extreme examples of protein dynamics: unfolding and/or folding of an entire protein, which can take times varying from microseconds to several seconds or minutes.^6^ Even for well structured proteins characterizing their motions requires the notion of an energy landscape.^7^

Decades after the first determinations of protein structures it emerged that many proteins in their functional state, unlike hemoglobin, contain regions which continuously sample widely different conformations. The prevalence of such ‘intrinsically disordered regions’ created a new field within protein science and made it overwhelmingly obvious that the structure–function dogma must embrace dynamics.^8,9^ Appreciating the dominant role of structural heterogeneity manifested in these proteins has led to much new insight into the molecular mechanisms of biology. A range of functional characteristics is associated precisely with the lack of a unique structure: the structural diversity enables an increased interaction capacity with a repertoire of partners. The complexity of the landscape makes possible the complex logical operations needed in the regulation and coordination of different signaling pathways.^10^ We now know that functional proteins as well as their complexes display a continuum of landscape patterns, ranging from those having nearly well-defined structures to those dominated by very fast-exchanging conformations.

**Figure 1:**
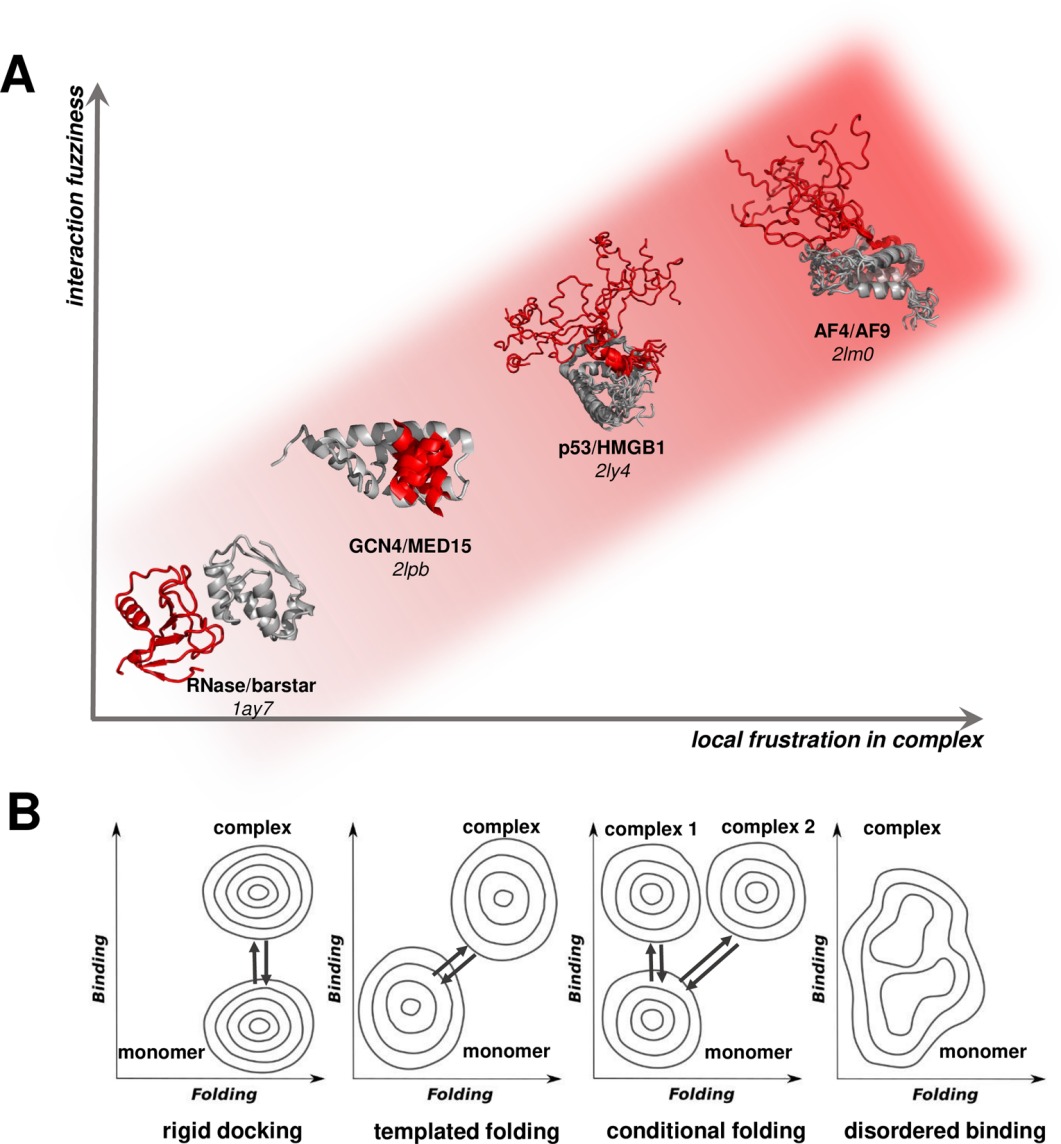
(A) Local frustration of the bound complex correlates to protein interaction fuzziness. Interactions between the two partners exhibit minimal frustration in rigid docking, which is coupled to moderate conformational changes upon interactions (represented by the RNase/barstar complex) and high frustration in disordered binding, when the bound partners are conformational heterogeneous in the complex (represented by the AF4/AF9 complex). Templated and conditional foldings, which are accompanied by a transition from disordered to ordered forms upon binding (represented by TADs of transcription factors GCN4 and p53), have intermediate local frustration, reflecting that the interactions of the folded elements are suboptimal. Indeed, in the case of conditional folding, the same protein region may remain disordered in complex with other proteins. (B) Folding and binding energy landscapes. The contour plots illustrate how folding frustration relates to interaction fuzziness by showing the energy landscapes in the free state (monomer) and the bound state (complex) along the folding and binding coordinates. In rigid docking, interactions take place between folded partners, resulting in a structured bound complex. In templated folding, the disordered partner(s) adopt a well-defined structure upon binding. Similar to templated folding, conditional folding may result in a structured complex upon binding with some partners (complex 2), but folding may not be induced upon assembly with other partners (complex 1). In the case of disordered binding, the energy landscapes of the monomeric and bound states overlap, as no folding takes place upon binding.

Here we discuss how protein structural ensembles form along the full dynamical continuum and how these ensembles have been evolutionary selected to satisfy a variety of cellular requirements. Our discussion is informed by merging two concepts: the frustration concept, which arose in statistical physics and has been developed to quantify conformational variations in structured proteins,^11^ and fuzziness, which captures functional heterogeneity seen so often in protein interactions^1^ (Figure 1). Both concepts are deeply related to the ruggedness of the free energy landscape, whose quantitative analysis has formed the basis of the modern unified theory of protein folding and assembly.

## 2 Protein Folding–Funneled Energy Landscapes

Protein folding is the process whereby a polypeptide chain is self-guided spontaneously to its native, functional conformation. Levinthal pointed out that there is not enough time for a polypeptide to sample all the conformations to find the most stable one.^12^ Originally, folding was conceived as a specific sequential mechanism characterized by the accumulation of partly folded intermediates, occurring through a stepwise folding, with local elements displaying a preformed structure progressively colliding to build the native state. This view was challenged by the discovery of globular domains capable of folding via an all-or-none two-state reaction.^13^ In such cases, folding must be a highly cooperative reaction, involving concurrent formation of secondary and tertiary interactions, with no detectable intermediates.^14−16^ These experimental observations could be understood using statistical mechanical energy landscape theory.^17,18^ This theory resolved the Levinthal paradox by introducing the notion of a “folding funnel”.

The consequences and implications of the funneled energy landscape theory have been extensively analyzed and reviewed over the years as the theory has continued to develop.^19−21^ From a mechanistic perspective, one of the most important predictions arising from the funneled energy landscape theory is that the strong energetic bias toward the native conformation would correspond to a transition state ensemble for folding made up of mostly native-like structural features. This observation has been validated on different protein systems, whose transition state ensemble of folding can be described as a large set of structures all stabilized by only a fraction of the interactions in the native state.^22−24^ More to the point, comparative studies on homologous proteins have revealed that these structural features arising from the protein topology are very robust, and proteins sharing the same structure but quite different sequences have been shown to fold via structurally similar transition state ensembles.^24−26^ These transition state ensembles are generally well predicted using models with perfectly funneled landscapes.^19,27−29^

## 3 Conformational Variations–Frustration of the Landscape

The resolution of Levinthal’s paradox that accounts for cooperative two-state folding is that the energy landscape of proteins is only minimally frustrated. Instead of there being strong conflicts among the interactions between residues in the amino acid sequence, evolution has selected sequences such that there is a strong energetic bias toward the native conformation.^27,30^ But what is protein frustration? Colloquially, frustration is a condition that arises from the inability to fulfill several goals at once. In a physical system, there is frustration when each of the energetic interactions holding the protein together cannot be individually yet simultaneously minimized by a single conformation. The funneled energy landscape theory postulates that there is a strong energetic bias toward the native state in which these frustrated conflicts are largely absent. Natural proteins have been evolutionary sculpted by natural selection to be minimally frustrated. Many sequences can satisfy the principle of minimal frustration for the same structure, underlying the fact that proteins come in families. Statistical analysis shows that the result of random mutagenesis and selection on protein structure is quantitatively consistent with a highly funneled landscape.^31^

Proteins, however, have evolved not only to fold robustly but also to be able to “function”.^32^ An activity such as binding or catalytic action may be in conflict with the overall structural architecture.^33^ Therefore, the native bias must be compromised locally by competing interactions in protein folding, which sometimes leads to some non-native structures in the folding process. An example is the intermediate formed in the folding of Im7, a small, fast folding protein that not only must fold but also must rapidly bind to a toxin^34−36^. Non-native contacts are also sampled in the folding transition state, as experimentally observed for frataxin.^34^ The organization of the conformational space of globular proteins is nevertheless overall consistent with the principle of minimal frustration, implying the presence of converging routes eventually leading to the native state.^37^ For functional reasons, however, proteins may contain a significant portion of frustration, which perturbs their folding routes. Consequently, competing routes do sometimes emerge, and non-native alternative free energy minima will be populated along the folding pathway.^34,38^ In our view, these examples provide exceptions that, however, corroborate the generally funnel-like nature of protein folding landscapes.

## 4 Templated Folding–Plasticity of Folding Pathways

Globular proteins usually attain a low degree of frustration finally by having a well-defined tertiary structure. However, increasing the frustration level promotes the population of new conformational substates, which results in more heterogeneous ensembles. Under such conditions, protein function can exploit then the ability to visit many conformational substates whose population can be modulated by other partners. Structural diversity can be manifested in many different ways: having different secondary structure conformations with similar probabilities;^39,40^ transiently populated conformations of flexible linkers, which control the arrangements of globular domains;^41^ switching between folded and unfolded states;^42^ as well as almost random seeming heteropolymers that interconvert between many different conformations.^43^ Proteins possessing such very broad structural ensembles are often denoted as ‘intrinsically disordered’. The native state of these proteins is a conformational ensemble, in contrast to a distinguished well-folded conformation.^39^ Almost all natural proteins do, in fact, have some degree of frustration, sampling a continuum between order (low frustration) and disorder (high frustration).^44^

In principle, through interaction of intrinsically disordered regions with other partner proteins, proteins may decrease their local frustration. In a binding-induced folding process, highly frustrated disordered proteins adopt a well-defined conformation once bound^45,46^ (Figure 1). In contrast to folding on highly funneled landscapes which often starts in clear locations,^14−16^ such partner-assisted folding often may begin from several nuclei, where the physiological partner controls (“templates”) the transition state of the folding reaction.^40,47−49^ Thus, the transition state of templated folding is liable to change with the experimental or cellular conditions.

A typical signature of frustration in protein folding lies in the presence of transient non-native interactions along the major folding pathway. This effect has been observed in several different protein systems including the aforementioned Im7, the designed and overstabilized Top7,^50,51^ alpha 3D,^52^ and frataxin.^34^ In these cases, large roughness of the folding energy landscape leads to multiple qualitatively different competing pathways. Different binding partners may tune the folding trajectories, so the distributions of the nucleation may differ. Consequently, although templated folding results in well-defined structures in the bound state, there is often considerable variability in the binding-induced folding process.

## 5 Fuzzy Binding–Heterogeneity in Bound Complexes

Proteins often interact in a context-dependent manner, denoted as fuzzy binding.^53^ Intrinsically disordered proteins, in particular, may adopt folded structures in some of their complexes, while they remain disordered when interacting with other partners,^54^ a phenomenon that is termed conditional folding (Figure 1). Such transitions may be also induced in the bound complex by post-translational modifications or can arise further via interactions with additional partners.^55,56^ The binding can be so fuzzy so that different ordered structures are populated in the bound state, as exemplified by the p53 C-terminal region.^57^ In this manner, fuzzy binding leads to a capability to interact in a versatile fashion with a defined but large set of partners. The phenomenon of fuzzy binding also suggests that templated folding can result in frustrated structures in the bound state. This may lead to conditional folding, when both ordered and disordered structures are populated in the bound state (Figure 1).^58,59^

**Figure 2:**
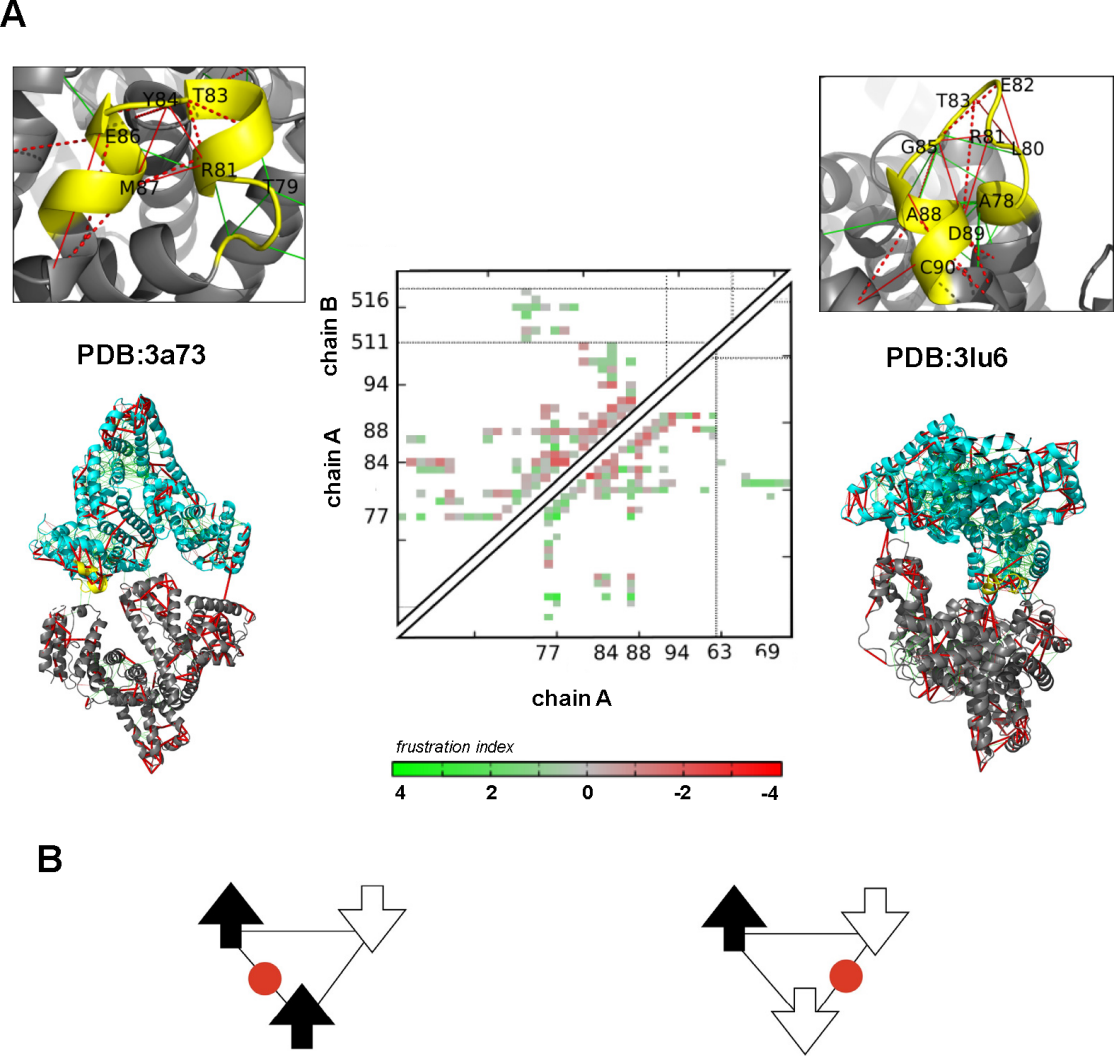
Specificity in a frustrated complex leads to different contact patterns with different partners. (A) Human serum albumin binds the natural ligand prostaglandin (PDB: 3a73) and a drug compound (PDB: 3lu6) in alternative binding modes via an extensive set of aromatic and electrostatic interactions. These contacts and the level of frustration can vary depending on the target and fine-tune affinities with different ligands. Local frustration values associated with the contacts are shown in Table S1. Local frustration patterns of thc are shown with minimally frustrated interactions in green and highly frustrated interactions in red. The fuzzy region (77–90 residues) is displayed as a yellow backbone. In the contact map, minimally frustrated contacts are shown in green, highly frustrated contacts are shown in red, and neutral contacts are shown in gray (as shown in the horizontal bar below the contact map). Some contacts are not displayed for better visualization. (B) Frustration in magnets also leads to alternative spin arrangements. A triangular ferromagnetic lattice is represented with the spins as arrows, and the favorable antiferromagnetic interactions are represented with lines. There is no way to arrange the spins such that they are all satisfied; in any case, an unfavorable interaction remains (red dot). This triangular lattice is thus energetically frustrated.

Recently, the energy landscape theory was applied to assess the level of local frustration in protein complexes, which are formed by templated folding.^3^ It has been shown that partner interactions reduce but do not always entirely eliminate local frustration in the final complexes of disordered protein regions. These results indicate that binding-induced folding often results in suboptimal interactions both at the binding interface as well as in the structured part of the protein. Protein regions which adopt ordered structures in a context-dependent manner also display frustrated patterns in their protein complexes.^3^ The energy landscape theory has also illuminated how selectivity is achieved in frustrated bound complexes. Owing to the presence of multiple suboptimal conformations, binding to different partners results in distinct frustration patterns^3^ (Figure 2, Table S1). Thus, the frustrated conformational energy landscape in the bound state seems to provide a functional advantage by allowing an increased interaction capacity with a limited set of partners.

The sequence properties that lead to the emergence of fuzzy binding have been recently elaborated. Interaction motifs, with distinguished composition and physicochemical features as compared to their flanking regions, will undergo disorder-to-order transition upon binding and fold in a partner induced manner.^60^ In contrast, protein motifs similar to their embedding regions likely remain disordered in the bound state.^60^ Within this framework, protein complexes sample a dynamical continuum from well-defined, ordered bound states to highly heterogeneous, disordered bound states, and the different modes of binding can be quantified by the conformational entropy. Frustration of the energy landscape in the bound state leads to variation between these different binding modes. Based on the local sequence biases with different possible binding sites, the pool of possible binding modes leading to fuzziness can also be quantified.^53^ Frustration in the binding landscape also may stem from non-native interactions as observed for c-Myb/KIX^61−63^ or ACTR/NCBD.^64,65^

All these alternative binding modes expand the functional repertoire of proteins, as the relative populations of the different conformational substates can now be shifted according to the cellular milieu.^66^

## 6 Fuzzy Interactions–Evolutionary Implications

The possibility of expanding the functional repertoire through frustrated interactions suggests an interesting evolutionary mechanism. The ability of any system to respond to a selection pressure demands its constituents to be mutable and malleable. In the case of a cell, optimization of fitness is a constantly ongoing process in which conditions change over both evolutionary and ecological time scales and where surviving lineages manage to adapt via changes in expression levels of proteins as well as the protein sequences themselves. From a protein structure point of view, it is clear that both extremely well-folded as well as highly dynamic proteins are vital for life. It is not clear to what extent ‘new’ folded proteins arise during evolution, but it is likely that the majority of folded proteins today are descendants of ancient folded structures. On the other hand, short linear or peptide interaction motifs consisting of a few up to a dozen amino acid residues recurrently arise de novo, as demonstrated for virus-host protein–protein interactions.^67^ The evolutionary history of the interaction between a longer disordered region called CID from the transcriptional coactivator ACTR and the NCBD domain of CBP/p300 has recently been investigated.^61,65^ Here, a likely evolutionary scenario is that the ancestral interaction involved a weaker binding that was under positive selection and that a few key mutations in NCBD finally yielded a functional affinity. Interestingly, structural data^4^ suggested that the plasticity of the disordered CID could relieve frustration and compensate for apparently detrimental point mutations.

**Figure 3:**
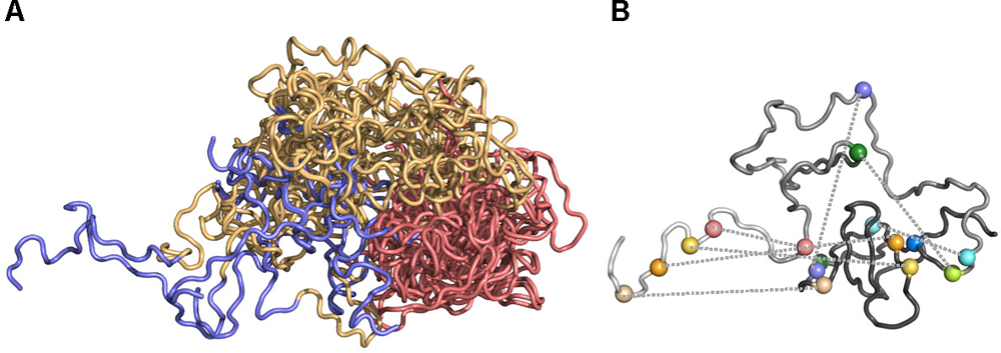
Fuzzy interactions with coevolutionary signals. (A) N-terminal regulatory region of c-Src, where the SH4 (teal), Unique (light orange), and SH3 (salmon) domains form a compact but highly dynamic, supramodular structure due to alternative long-range interactions. (B) Residues belonging to different domains with high coevolutionary probability on a single conformer. The three domains are shown by different shades of gray, which is gradually increasing from SH4 toward SH3. Residue pairs are displayed by identical colors.

The idea that structural pliability gives an evolutionary edge with respect to the emergence of new protein–protein interactions is appealing. Along these lines, it is likely that initial promiscuous interactions between a disordered region and a protein domain may explore alternative conformations. While many solved complexes between disordered regions and folded domains are structurally well-defined, like that for CID/NCBD, there are examples of positive selection for interactions with fuzzy complexes.^68,69^ Coevolutionary analysis provides a powerful method to identify intermolecular interaction partners as well as long-range intramolecular contacts within a protein. Analysis of coevolving residues has demonstrated that the supramodular structure of the nonreceptor tyrosine kinase c-Src involves intramolecular contacts between two domains (Unique and SH3),^70^ an interaction, which is structurally heterogeneous as shown by biophysical methods (Figure 3). Another example is the fuzzy interaction between S100B and ribosomal S6 kinase, which is conserved among vertebrates.^71^ Thus, in a cell, disordered protein regions are constantly being exposed to various other proteins and engage in numerous promiscuous binding events, also referred to as quinary interactions.^72^ Whenever such interactions are advantageous to the organism, they might become fixed in the population by positive selection. The original interaction was likely frustrated and explored multiple conformations, but then depending on the evolutionary dice and functional restraints, further point mutations might lead the complex to evolve into a more minimally frustrated and structurally well-defined complex, like that between CID and NCBD. Alternatively, the complex may remain locally frustrated and structurally heterogeneous, fuzzy, as seen for Unique/SH3. It is clear that selection has generated a wide spectrum of local frustration levels in natural protein–protein interactions, resulting in well-defined complexes as well as sometimes structurally heterogeneous complexes.

## 7 A Unified Model: Frustration vis-à-vis Fuzziness

Frustration implies deviations from the perfect funnel-like energy landscape, often resulting in conformational heterogeneity of native proteins. Ruggedness of the landscape is caused by the suboptimal interactions that take place through non-native contacts, and thus we use it in thermodynamic terms. Multiple, minimalistic motifs coupled to non-native interactions may compromise folding. Ruggedness of the binding energy landscape can also arise from suboptimal, often redundant interaction motifs, for example, in low-complexity protein regions. Frustration results in alternative binding modes, which imparts plasticity to the templated folding mechanism and can enhance adaptability to a range of partners in a versatile form. Thus, ruggedness of the binding landscape leads to fuzzy binding and variations between many different interaction modes. Similar to frustration in free proteins, fuzziness of protein complexes also expands the functional repertoire to achieve fine-tuned, context-dependent regulation.^73,74^

Here we have shown that frustration and fuzziness are parallel concepts, aspects of suboptimal interactions of the energy landscape (Figure 1). This can be illustrated by the N-terminal regulatory region of c-Src, where the SH4, Unique, and SH3 domains form a compact but highly dynamic, supramodular structure due to alternative long-range interactions (Figure 3).^70^ Residues mediating fuzzy interactions are well-conserved and often display coevolutionary signatures. This example illustrates the way fuzziness originating in frustrated contacts can control signaling specificity.

## 8 Conclusions

Several observations suggest that the contrasting requirements of physical folding and biological function may give rise to energetic frustration in natural proteins. Conformational heterogeneity promotes adaptability which itself may often be under evolutionary selection. Landscape ruggedness can be modulated by interactions with physiological partners. Suboptimal contacts with the partner often lead to fuzzy binding and give protein complexes with partner-specific, alternative binding modes. Although the concept of local frustration has been applied to folding and function of globular proteins, while fuzziness has mostly been discussed in the area of protein interactions and assemblies, these are really two sides of the same coin. Both frustration and fuzziness emerge from common physical principles of the energy landscape and can be described by a common formalism. We feel this realization can open new perspectives unifying protein folding and assembly with biological function.

## Supporting Information Available

Table S1, frustration index for contacts displayed in Figure 2 (PDF)
