# Supplementary material for: Fuzziness and Frustration in the Energy Landscape of Protein Folding, Function, and Assembly
Source: Acc Chem Res. 2021 Feb 8;54(5):1251–9. doi: 10.1021/acs.accounts.0c00813 (PMC8023570; doi:10.1021/acs.accounts.0c00813)
Supplement: Supplementary file 1 — ar0c00813_si_001.pdf [file ar0c00813_si_001.pdf]

Fuzziness and frustration in the energy landscape of protein folding, function and assembly

Stefano Gianni[1*], María Inés Freiberger[2], Per Jemth[3], Diego U. Ferreiro[2], Peter G. Wolynes[4*], Monika Fuxreiter[5,6*]

[1] *Istituto Pasteur - Fondazione Cenci Bolognetti, Dipartimento di Scienze Biochimiche "A. Rossi Fanelli" and Istituto di Biologia e Patologia Molecolari del CNR, Sapienza Università di Roma, 00185, Rome, Italy*

[2] *Protein Physiology Lab, Departamento de Química Biológica, Facultad de Ciencias Exactas y Naturales, Universidad de Buenos Aires-CONICET-IQUIBICEN, 1428, Buenos Aires, Argentina*

[3] *Department of Medical Biochemistry and Microbiology, Uppsala University, Husargatan 3, SE-75123 Uppsala, Sweden*

[4] *Center for Theoretical Biological Physics, Rice University, 6500 Main St, Houston, Texas 77251-1892*

USA

[5]*MTA-DE Laboratory of Protein Dynamics, Department of Biochemistry and Molecular Biology, University of Debrecen, Nagyerdei krt 98, H-4032, Debrecen, Hungary*

[6]*Department of Biomedical Sciences, University of Padova, Via Ugo Bassi 58/B, 35131 Padova, Italy*

Correspondence: stefano.gianni@uniroma1.it, pwolynes@rice.edu, monika.fuxreiter@unipd.it

Table S1: Frustration index for contacts displayed on Figure 2.
Frustration index was defined as [1]

$$F_i = \frac{E_i^{T,N} - \langle E_{i'}^{T,U} \rangle}{\sqrt{1/N \sum_{k=1}^{n} (E_{i'}^{T,U} - \langle E_{i'}^{T,U} \rangle)^2}}.$$

**3a73**					**3lu6**				
#Res1	#Res2	Chain Res1	Chain Res2	Frustration_Index	#Res1	#Res2	Chain Res1	Chain Res2	Frustration_Index
30	84	A	A	-1.081	34	87	A	A	-1.008
33	84	A	A	-1.467	75	87	A	A	-1.022
81	84	A	A	-1.987	80	82	A	A	-2.347
81	85	A	A	-1.633	80	83	A	A	-1.256
81	88	A	A	-1.085	81	85	A	A	-1.093
82	84	A	A	-1.899	81	88	A	A	-1.41
82	88	A	A	-1.154	82	84	A	A	-1.323
84	86	A	A	-1.083	83	88	A	A	-1.147
85	88	A	A	-1.309	85	88	A	A	-1.423
86	89	A	A	-1.652	86	89	A	A	-1.583
87	91	A	A	-1.586	87	91	A	A	-1.013
87	101	A	A	-1.591	87	101	A	A	-1.706
88	92	A	A	-1.143	88	92	A	A	-1.25
89	92	A	A	-1.824	89	92	A	A	-1.621
					89	101	A	A	-1.825

References

(1) Ferreiro, D. U.; Komives, E. A.; Wolynes, P. G. Frustration in biomolecules. *Quarterly reviews of biophysics* **2014**, *47*, 285-363.
